# Fission Yeast Scp3 Potentially Maintains Microtubule Orientation through Bundling

**DOI:** 10.1371/journal.pone.0120109

**Published:** 2015-03-13

**Authors:** Kanako Ozaki, Yuji Chikashige, Yasushi Hiraoka, Tomohiro Matsumoto

**Affiliations:** 1 Graduate School of Biostudies, Kyoto University, Kyoto, Kyoto, Japan; 2 Advanced ICT Research Institute Kobe, National Institute of Information and Communications Technology, Kobe, Hyogo, Japan; 3 Graduate School of Frontier Biosciences, Osaka University, Suita, Osaka, Japan; 4 Radiation Biology Center, Kyoto University, Kyoto, Kyoto, Japan; Cancer Research UK London Research Institute, UNITED KINGDOM

## Abstract

Microtubules play important roles in organelle transport, the maintenance of cell polarity and chromosome segregation and generally form bundles during these processes. The fission yeast gene *scp3* ^+^ was identified as a multicopy suppressor of the *cps3-81* mutant, which is hypersensitive to isopropyl N-3-chlorophenylcarbamate (CIPC), a poison that induces abnormal multipolar spindle formation in higher eukaryotes. In this study, we investigated the function of Scp3 along with the effect of CIPC in the fission yeast *Schizosaccharomyces pombe*. Microscopic observation revealed that treatment with CIPC, *cps3-81* mutation and *scp3* ^+^ gene deletion disturbed the orientation of microtubules in interphase cells. Overexpression of *scp3* ^+^ suppressed the abnormal orientation of microtubules by promoting bundling. Functional analysis suggested that Scp3 functions independently from Ase1, a protein largely required for the bundling of the mitotic spindle. A strain lacking the *ase1* ^+^ gene was more sensitive to CIPC, with the drug affecting the integrity of the mitotic spindle, indicating that CIPC has a mitotic target that has a role redundant with Ase1. These results suggested that multiple systems are independently involved to ensure microtubule orientation by bundling in fission yeast.

## Introduction

Microtubules are polymers formed by the association of α- and β-tubulin dimers, and they have different roles in each phase of the cell cycle [1–4]. In interphase, cellular processes such as organelle transport, maintenance of cell polarity as well as cell shape depend on microtubules as a component of the cytoskeleton [5–7]. In addition, microtubules play an essential role as spindle machinery for chromosome segregation and cytokinesis during mitosis [8,9]. These functions of microtubules can be targets of anticancer drugs and herbicides that block normal mitotic spindle organization, inhibit cell cycle progression and induce chromosome missegregation [10–13].

However, proper regulation of the dynamic and reversible process of microtubule polymerization/depolymerization between interphase and mitosis depends on not only the function of tubulin itself but also on other regulators and binding proteins. For instance, +Tips have roles in capping proteins of the microtubule plus end, and microtubule organizing center (MTOC) and end-directed motor proteins are also important for the mitotic spindle [14–20]. Although these regulators bind microtubules directly, the mechanisms regulating microtubules, including those of indirect regulators, have only partially been elucidated.

In general, microtubules form anti-parallel bundles *in vivo*. One regulator of microtubule bundling is Ase1, a protein first identified in budding yeast as a conserved microtubule-associated protein [21,22]. In human cells, a homolog of Ase1 called PRC1 was identified as a mitotic CDK substrate required for the maintenance of the spindle mid-zone and cytokinesis [23–25]. In fission yeast, Ase1 is also a target of CDK in metaphase and acts with Klp9 in spindle formation [26–28]. Microtubule bundles are important for cell morphology in interphase and normal mitotic spindle formation.

CIPC (isopropyl N-3-chlorophenylcarbamate) is an herbicide that has been shown to induce multipolar spindles in mitosis [29–33] Although previous studies have reported the isolation of mutants hypersensitive to CIPC and the cloning of their suppressors in fission yeast, the effect of the drug remains unclear [34,35]. In this study, we attempted to examine the effects of CIPC. Observation of microtubules revealed that the drug likely affects bundling. The *cps3–81* mutant, which is hypersensitive to CIPC, and a strain lacking the *scp3* ^+^ gene, a multicopy suppressor of the *cps3–81* mutant, exhibited abnormal microtubule morphology indicative of the loss of bundling. We further showed that Scp3 and Ase1 function independently in the maintenance of microtubule bundling in interphase and that Ase1, but not Scp3, also plays an important role in mitotic spindle organization.

## Materials and Methods

### Yeast strains, media, drug and transformation

The *Schizosaccharomyces pombe* strains used in this study are listed in Table 1. The strains were grown in YES (yeast extract with supplement) medium or EMM synthetic minimal medium with appropriate nutrient supplements, as described by Moreno et al [36]. Yeast transformation was carried out by the lithium acetate method [37–39]. CIPC (Sigma) in DMSO was added to the media. For microscopic observation, cells were cultured in the appropriate medium containing CIPC for 5 hours.

**Table 1:** Strains used in this study.

Strain	Genotype	Source
SP6	*h* ^*-*^ *leu1–32*	lab stock
OZK-1	*h* ^*+*^ *leu1–32 cps3–81*	This study
OZK-2	*h* ^*-*^ *leu1–32 ase1*::*kan* ^*R*^	This study
OZK-3	*h* ^*+*^ *leu1–32 cps3–81 ase1*::*kan* ^*R*^	This study
OZK-4	*h* ^*-*^ *leu1–32 scp3*::*kan* ^*R*^	This study
OZK-5	*h* ^*-*^ *leu1–32 pREP1*	This study
OZK-6	*h* ^*-*^ *leu1–32 pREP1-scp3* ^*+*^	This study
OZK-7	*h* ^*-*^ *leu1–32 pREP41-scp3* ^*+*^	This study
OZK-8	*h* ^*-*^ *leu1–32 pREP81-scp3* ^*+*^	This study
OZK-9	*h* ^*+*^ *leu1–32 GFP-atb2-kan* ^*R*^ *sad1-mCherry-kan* ^*R*^	This study
OZK-10	*h* ^*+*^ *leu1–32 GFP-atb2-kan* ^*R*^ *sad1-mCherry-kan* ^*R*^ *pREP1*	This study
OZK-11	*h* ^*+*^ *leu1–32 GFP-atb2-kan* ^*R*^ *sad1-mCherry-kan* ^*R*^ *pREP1-scp3* ^*+*^	This study
OZK-12	*h* ^*+*^ *leu1–32 GFP-atb2-kan* ^*R*^ *sad1-mCherry-kan* ^*R*^ *pREP41-scp3* ^*+*^	This study
OZK-13	*h* ^*+*^ *leu1–32 GFP-atb2-kan* ^*R*^ *sad1-mCherry-kan* ^*R*^ *pREP81-scp3* ^*+*^	This study
OZK-14	*h* ^*-*^ *leu1–32 scp3*::*kan* ^*R*^ *GFP-atb2-kan* ^*R*^ *sad1-mCherry-kan* ^*R*^	This study
OZK-15	*h* ^*-*^ *leu1–32 scp3*::*kan* ^*R*^ *GFP-atb2-kan* ^*R*^ *sad1-mCherry-kan* ^*R*^ *pREP1*	This study
OZK-16	*h* ^*-*^ *leu1–32 scp3*::*kan* ^*R*^ *GFP-atb2-kan* ^*R*^ *sad1-mCherry-kan* ^*R*^ *pREP81-ase1* ^*+*^	This study
OZK-17	*h* ^*-*^ *leu1–32 scp3*::*kan* ^*R*^ *lys1* ^*+*^ *-Pnda3-GFP-atb2 sad1-mCherry-kan* ^*R*^	This study
OZK-18	*h* ^*+*^ *leu1–32 cps3–81 GFP-atb2-kan* ^*R*^ *sad1-mCherry-kan* ^*R*^ *pREP1*	This study
OZK-19	*h* ^*+*^ *leu1–32 cps3–81 GFP-atb2-kan* ^*R*^ *sad1-mCherry-kan* ^*R*^ *pREP81-scp3* ^*+*^	This study
OZK-20	*h* ^*-*^ *leu1–32 ase1*::*kan* ^*R*^ *lys1* ^*+*^ *-Pnda3-GFP-atb2 sad1-mCherry-kan* ^*R*^	This study
OZK-21	*h* ^*+*^ *leu1–32 ase1*::*kan* ^*R*^ *scp3*::*kan* ^*R*^ *lys1* ^*+*^ *-Pnda3-GFP-atb2 sad1-mCherry-kan* ^*R*^	This study
OZK-22	*h* ^*-*^ *leu1–32 ase1*::*kan* ^*R*^ *lys1* ^*+*^ *-Pnda3-GFP-atb2 sad1-mCherry-kan* ^*R*^ *pREP1*	This study
OZK-23	*h* ^*-*^ *leu1–32 ase1*::*kan* ^*R*^ *lys1* ^*+*^ *-Pnda3-GFP-atb2 sad1-mCherry-kan* ^*R*^ *pREP81-scp3* ^*+*^	This study
OZK-24	*h* ^*-*^ *leu1–32 sad1-mCherry-kan* ^*R*^ *pREP1*	This study
OZK-25	*h* ^*-*^ *leu1–32 sad1-mCherry-kan* ^*R*^ *pREP81-scp3* ^*+*^ *-GFP*	This study
OZK-26	*h* ^*+*^ *leu1–32 ura4-D18 scp3* ^*+*^ *-GFP-ura4* ^*+*^	This study
OZK-27	*h* ^*-*^ *leu1–32 ura4-D18 sad1-mCherry-kan* ^*R*^ *scp3* ^*+*^ *-GFP-ura4* ^*+*^ *pREP1*	This study
OZK-28	*h* ^*-*^ *leu1–32 ura4-D18 GFP-atb2-kan* ^*R*^ *sad1-mCherry-kan* ^*R*^ *scp3* ^*+*^ *-GFP-ura4* ^*+*^	This study
OZK-29	*h* ^*+*^ *leu1–32 mto1-GFP-kan* ^*R*^ *sad1-mCherry-kan* ^*R*^	This study
OZK-30	*h* ^*+*^ *leu1–32 scp3*::*kan* ^*R*^ *mto1-GFP-kan* ^*R*^ *sad1-mCherry-kan* ^*R*^	This study
OZK-31	*h* ^*+*^ *leu1–32 alp4-GFP-kan* ^*R*^ *sad1-mCherry-kan* ^*R*^	This study
OZK-32	*h* ^*+*^ *leu1–32 scp3*::*kan* ^*R*^ *alp4-GFP-kan* ^*R*^ *sad1-mCherry-kan* ^*R*^	This study
OZK-33	*h* ^*+*^ *leu1–32 ase1-GFP-kan* ^*R*^ *sad1-mCherry-kan* ^*R*^	This study

### Plasmids

Enzymes were used as recommended by the suppliers (NEB, TOYOBO, Takara Shuzo and Promega). *S*. *pombe* expression vectors pREP1, -41, and -81, which carry the thiamine-repressible *nmt1* promoter [40], were used for overexpressing *scp3* ^+^ and *ase1* ^+^. The *scp3* ^+^ and *ase1* ^+^ gene ORFs were amplified using the KOD-Plus PCR kit (TOYOBO) with a pair of primers carrying an *NdeI* and a *NotI* site. The PCR products were digested with *NdeI* and *NotI* and cloned into each plasmid.

### Construction of Scp3-GFP

A homologous recombination-based method was used to tag endogenous Scp3 with GFP (Green Fluorescent Protein) at its carboxyl terminus. The *scp3* ^+^ ORF, except for the first methionine and terminal codon, was amplified using the KOD-Plus PCR kit (TOYOBO) with a pair of primers carrying *SalI* and *NotI* sites. The PCR product was digested with *SalI* and *NotI* and cloned into the plasmid, with the inserted GFP fragment located in frame at the carboxyl terminus. For ectopic expression of Scp3 tagged with GFP, the ORF of the *scp3* ^+^ gene, except for the terminal codon, was amplified using the KOD-Plus PCR kit (TOYOBO) with a pair of primers carrying *SalI* and *NotI* sites and cloned into the pREP81 plasmid, with the inserted GFP fragment located at the carboxyl terminus in frame.

### Fluorescence microscopy

Hoechst33342 (Nacalai tesque) was used to stain DNA in living cells expressing GFP-Atb2 and Sad1-mCherry from the native loci. Still images were acquired using a laser-scanning microscope (DM5500B; Leica). The objective lens and CCD camera were an oil-immersion HCX PL APO 100 × /1.40 lens (Leica) and ORCA-ER C4742–80 digital camera (Hamamatsu photonics), respectively. The images were processed with IP lab software (BD).

For time-lapse observation, cells expressing GFP-Atb2 and Sad1-mCherry from the native locus were grown to mid-logphase in EMM medium supplied with leucine at 30°C, then cells were mounted in a microfluidic flow chamber for yeast (CELLASIC Y04C), and observed in EMM-supplied with leucine at 30°C. The flow pressure was 1 psi. Fluorescence microscope images were obtained by the DeltaVision microscope system (Applied Precision, Inc.) set up in a temperature-controlled room as previously described [41,42]. This microscope system is based on an inverted fluorescence microscope (IX71; Olympus) equipped with a charge-coupled device (CoolSNAP HQ2; Photometrics). The objective lens used was an oil immersion Plan-Apo 60 × NA = 1.4 lens (Olympus). A stack of nine slices (0.3 μm distance between planes) was projected with softWoRx software (Applied Precision, Inc.) using a maximum intensity method [41,42].

## Results and Discussion

### Effect of CIPC

**Fig 1:**
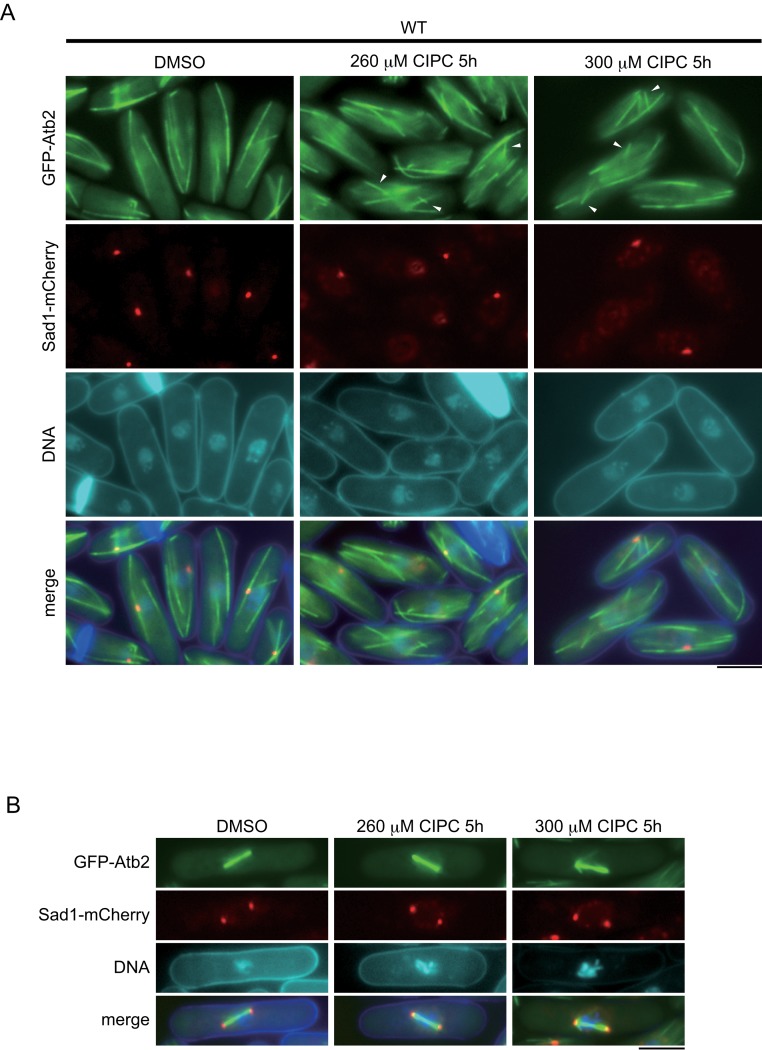
Microtubules in the wild-type strain treated with CIPC. (A) GFP-tagged α-tubulin (GFP-Atb2) as a microtubule marker and Sad1 tagged with mCherry (Sad1-mCherry) as an SPB marker were expressed from their native promoters. The cells were grown at 30°C and treated with 260 μM or 300 μM CIPC for 5 hours in EMM medium. DMSO was used as a solvent. Misoriented MTs are marked with arrowheads. The bar indicates 5 μm. (B) The wild-type strain in metaphase was observed in the presence of CIPC. The bar indicates 5 μm.

**Fig 2:**
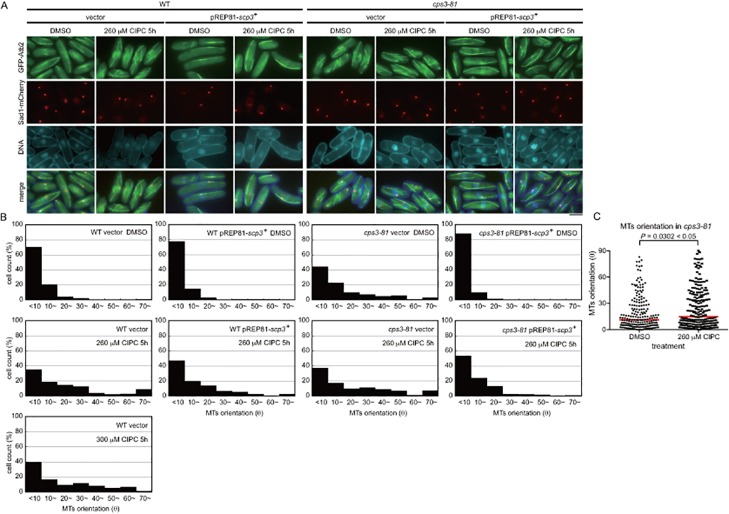
The effect of Scp3 overexpression. (A) Each strain expressing GFP-Atb2 and Sad1-mCherry, as in Fig. 1A, was first grown in EMM containing thiamine at 30°C. Thiamine was washed out to induce expression of the *scp3* ^+^ gene from the *nmt* promoter of pREP81. Each strain was cultured for 20 hours, and CIPC (260 μM) was added for 5 hours. The bar indicates 5 μm. (B) The angle between the long cell axis and each MT was measured for the analysis of microtubule orientation. More than 150 microtubules were observed under each condition. (C) The effect of CIPC to the *cps3–81* mutant was analyzed by Nonparametric Mann-Whitney *U* test. The *cps3–81* mutants transformed with an empty vector were grown in the presence or absence of CIPC for 5 hours were compared. The red lines are the median.

It was previously reported that isopropyl N-3-chlorophenylcarbamate (CIPC) induces abnormal multipolar spindle formation in higher eukaryotes [31]. We therefore examined the effect of the drug on fission yeast, focusing on the morphology of microtubules (MTs). Throughout this study we employed strains expressing α-tubulin tagged with GFP from the native *nda3* promoter integrated at the *lys1* locus or the native *atb2* promoter at the native locus for visualization of MTs (GFP-Atb2) [43–45]. Because the survival rate of the wild-type cells is more than 90% at a concentration of 250 μM of CIPC and drops to nearly 0% at 300 μM [34], we first observed cells incubated in the presence of the drug at a semi-lethal concentration (260 μM) for 5 hours. As shown in Fig. 1A, the MTs in the cells treated with the drug were misoriented. For a statistical analysis, we measured the angle (θ) between the long cell axis and each MT. The results (Fig. 2B) showed that although the angles between the cell axis and most of the MTs (approximately 70%) in the control cells were less than 10°, they were much larger in the cells treated with the drug at 260 μM. We next examined wild-type cells treated with the drug at a lethal concentration (300 μM) for 5 hours. Despite the increase in the dose of CIPC, we did not note an apparent difference in the MT morphology (Figs. 1A and 2B). Under both conditions, the mitotic spindle and SPB (spindle pole body), a structure equivalent to the centrosome, were morphologically normal (Fig. 1B). We also observed living cells by time-lapse microscopy and found cell cycle progression was not disturbed during mitosis (S1 Fig).

**Fig 3:**
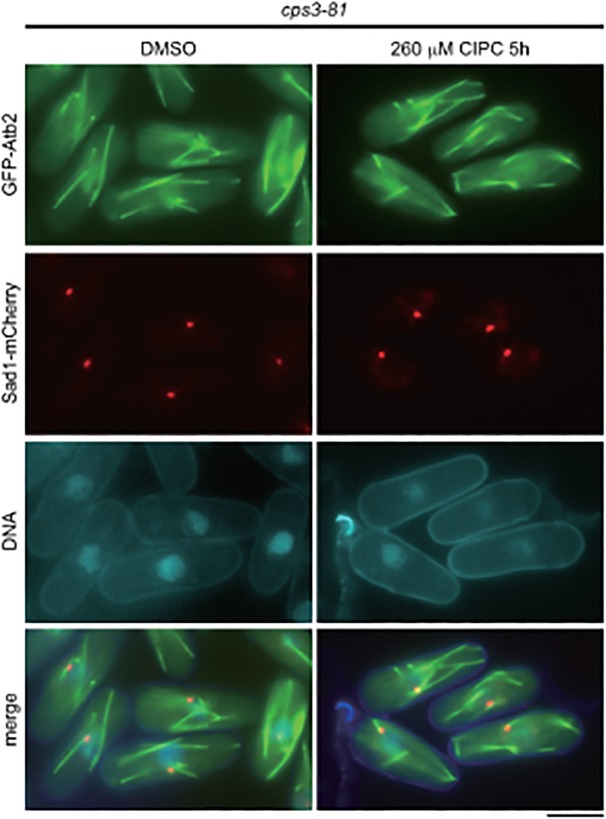
Microtubules in *cps3–81* treated with CIPC. The *cps3–81* mutant expressing GFP-Atb2 and Sad1-mCherry, as in Fig. 1A, was treated with DMSO (control) or 260 μM CIPC for 5 hours in EMM medium at 30°C. The bar indicates 5 μm.

The *cps3–81* mutant which is hypersensitive to CIPC, cannot grow in the presence of 260 μM of the drug [34,46]. Although the *cps3* ^+^ gene was genetically mapped very closed to the *arg1* locus (chromosome III), the gene has not been cloned to date [34]. As shown in Figs. 2B and 3, the MTs in the mutant were misoriented even when the cells were grown in the absence of CIPC. In the medium containing the drug at a lethal concentration (260 μM), the *cps3–81* mutant cells exhibited abnormal MT morphology that was slightly more prominent than that observed in the cells grown in the absence of the drug (Figs. 2B and 3). Our examination of the morphology of MTs in the cells failed to demonstrate why the increase in the dose of CIPC kills the cells. Nonetheless, it did indicate that CIPC largely affects the orientation of MTs during interphase in fission yeast.

### Characterization of the *scp3* ^+^ gene

**Fig 4:**
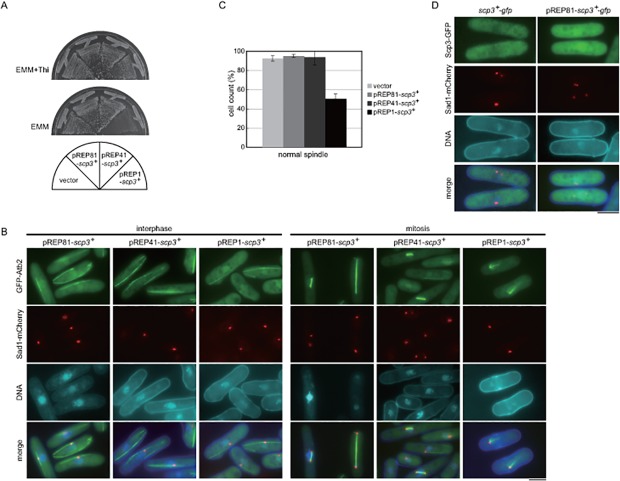
Localization of Scp3. (A) Each strain was grown on EMM medium with or without thiamine at 30°C for 5 days. (B) Microtubules in interphase or mitosis were observed in each strain. The cells were incubated in the absence of thiamine for 20 hours at 30°C. The bars indicate 5 μm. (C) Quantitative of normal mitotic spindle. (D) Cells expressing Scp3 tagged with GFP from the native promoter (left) or the promoter of pREP81 (right) were grown in EMM medium at 30°C. For induction of the *scp3* ^+^ gene, the cells were incubated in the absence of thiamine for 20 hours. Sad1-mCherry was used as a maker of SPB. The bars indicate 5 μm.

The *scp3* ^+^ gene was previously identified as a multicopy suppressor of the *cps3–81* mutant, which is hypersensitive to CIPC [46]. The gene encodes a protein of 583 amino acids, with a predicted molecular weight of 62.8 kDa and two zinc finger motifs starting at the 41^st^ and 70^th^ residues, respectively. As the *scp3* ^+^ gene was physically mapped on chromosome I, it is not allelic to the *cps3* ^+^ gene, which was mapped on chromosome III. We first confirmed that the *cps3–81* mutation is suppressed by the ectopic expression of the *scp3* ^+^ gene. *cps3–81* mutant cells ectopically expressing the *scp3* ^+^ gene from the *nmt* promoter of the pREP81 plasmid were able to grow on a medium containing CIPC at a concentration of 260 μM. Furthermore, MT observation indicated that *scp3* ^+^ overexpression corrected the abnormal morphology of MTs in the mutant (Fig. 2A and 2B). The ectopic expression of the *scp3* ^+^ gene from the *nmt* promoter of pREP81 also overcame the effect of the drug in wild-type cells (Fig. 2A and 2B). When *scp3* ^+^ expression was driven by the pREP1 promoter for a higher induction, growth retardation resulted in the wild-type strain (Fig. 4A). Microscopic observation indicated fewer interphase MTs and also aberrant mitotic spindles (Fig. 4B and 4C), suggesting that Scp3 promotes the bundling of MTs.

To determine the localization of Scp3, the *scp3* ^+^ gene tagged with GFP at the carboxyl terminus was expressed from the native promoter or overexpressed ectopically from the *nmt* promoter of the pREP81 plasmid. As shown in Fig. 4D, the majority of the fluorescence was found in the cytoplasm and nucleus. Because the signal intensity of Sad1-mCherry seemed to be reduced when Scp3-GFP was overexpressed (Fig. 4D), we tested whether overexpression of Scp3 affected the signal of Sad1-mCherry in a separate experiment. As shown in S2A Fig, observation of wild type cells and cells overexpressing Scp3-GFP on the same coverslip indicated that overexpression of Scp3-GFP did not affect the signal of Sad1-mCherry. The tagged construct, Scp3-GFP, was functional because MTs was not misoriented in the strain expressing it from the native locus (S2B and S2C Fig). We also tested the functionality of the tagged construct by examining the sensitivity to CIPC. As shown in S2D Fig, the strain expressing Scp3-GFP from the native locus was not sensitive to the drug, indicating that the tagged construct was functional.

**Fig 5:**
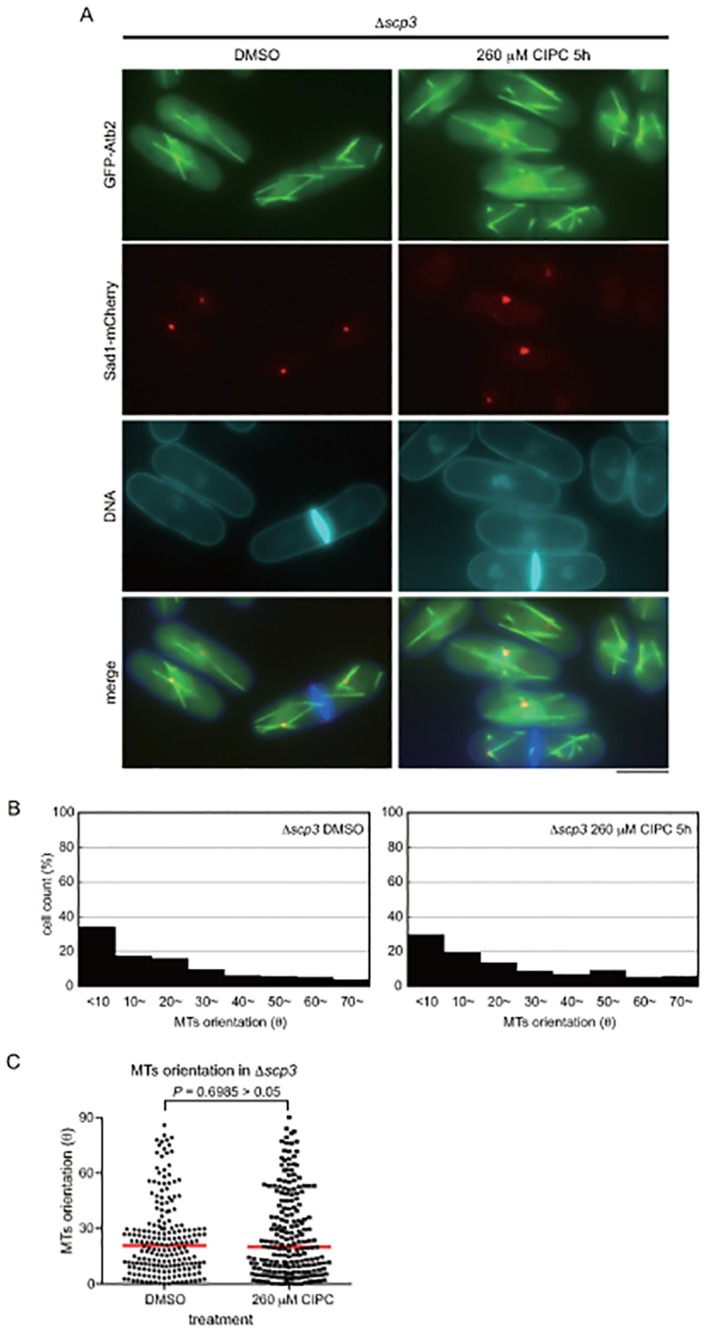
Abnormal microtubule orientation in Δ*scp3*. (A) The *∆scp3* strain expressing GFP-Atb2 and Sad1-mCherry from the native promoters was treated with DMSO or 260 μM CIPC for 5 hours in EMM medium at 30°C. The bar indicates 5 μm. (B) Microtubule orientation was analyzed as in Fig. 2B. (C) Nonparametric Mann-Whitney *U* test of (B). The red lines are the median.

The *scp3* ^+^ gene was replaced with the *ura4* ^+^ gene in a diploid strain, and a stable Ura4+ diploid (*scp3* ^+^::*ura4* ^+^ */*+) was examined by tetrad analysis. Most of the tetrads produced four viable spores in which the Ura4 marker segregated into 2:2, indicating that the *scp3* ^+^ gene is not essential for viability. Observation of MTs in a strain deleted for the *scp3* ^+^ gene (Δ*scp3*) revealed the misorientation of MTs (Fig. 5A). As shown in Fig. 5B and 5C, the drug did not cause any additional effects on the orientation of MT in the Δ*scp3* strain, suggesting that the drug might target Scp3.

These results suggest that Scp3 promotes the bundling of MTs. As Scp3 itself was not localized to MTs, it may play an indirect role in MT bundling.

### Interaction with *ase1* ^+^

**Fig 6:**
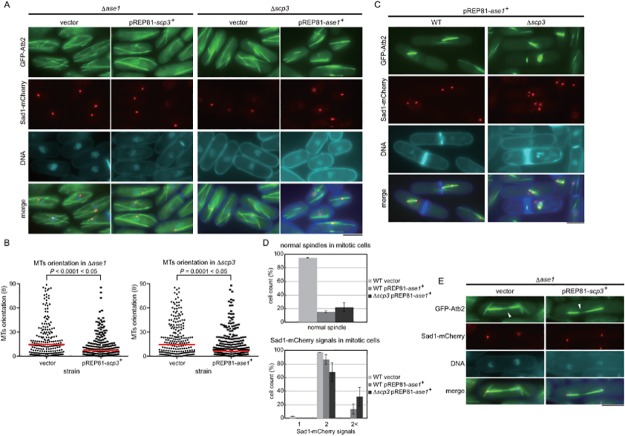
Relationship between Ase1 and Scp3. (A) Interphase MTs were observed in the Δ*ase1* strain overexpressing *scp3* ^+^ or the Δ*scp3* strain overexpressing *ase1* ^+^. Each gene was expressed from the *nmt* promoter of pREP81 in EMM medium at 30°C. (B) Nonparametric Mann-Whitney *U* test of (A). The red lines are the median. More than 150 microtubules were observed for each condition. (C) Mitotic cells overexpressing *ase1* ^+^ were observed. *ase1* ^+^ expression was induced for 18 hours. GFP-Atb2 expressed from the *nda3* promoter integrated at the *lys1* locus was used as a maker of microtubules and Sad1-mCherry for SPB. (D) Cells with normal spindle (upper) and with multiple SPBs (bottom) were counted for each strain. (E) *scp3* ^+^ was overexpressed in the Δ*ase1* strain. Aberrant mitotic spindles are marked with arrowheads. The bars indicate 5 μm.

Because the phenotype of the Δ*scp3* strain most resembled that caused by deletion of the *ase1* ^+^ gene [26,27], we attempted to investigate the functional relationship between Scp3 and Ase1. As shown in Fig. 6A and 6B, overexpression of *scp3* ^+^ or *ase1* ^+^ promoted the bundling of MTs in a strain lacking the other gene, suggesting that these two genes promote bundling independently. We also found that *ase1* ^+^ overexpression was toxic. The mitotic spindle often elongated beyond one of the SPBs in cells overexpression of *ase1* ^+^, likely due to the abnormal bundling of MTs composing the mitotic spindle (Fig. 6C and 6D). We also noticed that (1) mitotic cells overexpressing *ase1* ^+^ contained more than 2 SPBs, which could be caused by fragmentation of SPB and (2) this effect was more prominent in cells lacking *scp3* ^+^ (Fig. 6D), suggesting that Scp3 might be required for maintenance of the integrity of SPB when the spindle dynamics was perturbed.

*scp3* ^+^ overexpression did not suppress the collapse of the mitotic spindle occasionally caused by the loss of *ase1* ^+^ (Fig. 6E). Thus, Ase1 appears to function in MT bundling both in the nucleus and cytoplasm, whereas Scp3 acts largely in the cytoplasm. Orientation of MTs was also examined in the double mutant, Δ*ase1* Δ*scp3*. As shown in S3A and S3B Fig, the quantitative MT angle assays did not reveal the obvious additive effect between Δ*scp3* and Δ*ase1*, suggesting the following three possibilities. First, Scp3 and Ase1 are not fully independent and might have an overlapping function. Secondly, a third protein might compensate a loss of Scp3 and Ase1. Finally, the quantitative MT angle assays was exhausted and not sensitive enough to detect a slight additive effect caused in the double mutant. Future study is needed to reveal the functional relationship between the two proteins more precisely.

**Fig 7:**
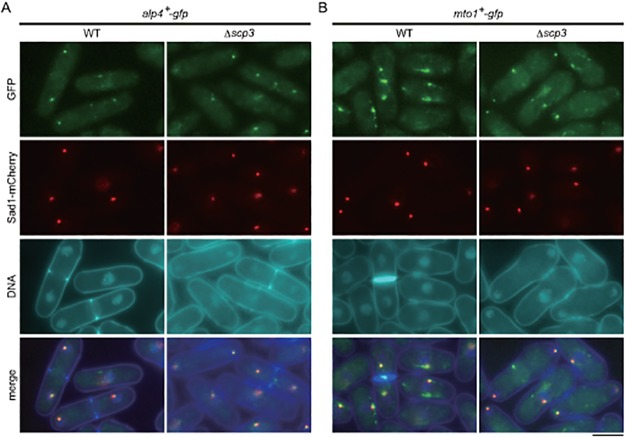
Components of MTOCs in Δ*scp3*. (A, B) Each protein tagged with GFP (Alp4 in A, Mto1 in B) was observed in EMM medium at 30°C. Sad1-mCherry was used as a marker of SPB. The bar indicates 5 μm.

**Fig 8:**
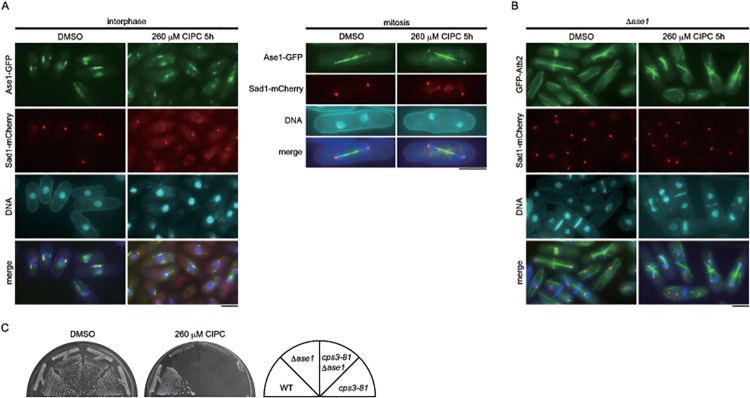
Relationship between Ase1 and CIPC. (A) Ase1 tagged with GFP was expressed from the native promoter in wild-type cultured in EMM medium containing DMSO or 260 μM CIPC for 5 hours at 30°C. Sad1-mCherry was used as an SPB maker. The bars indicate 5 μm. (B) The Δ*ase1* strain expressing GFP-Atb2 from the native *nda3* promoter integrated at the *lys1* locus and Sad1-mCherry from the native locus was cultured in EMM medium containing DMSO or 260 μM CIPC for 5 hours at 30°C. The bars indicate 5 μm. (C) Each strain was streaked on YES medium with or without CIPC (260 μM) and incubated at 30°C for 5 days.

Because the misorientation of MTs could be caused by a defect in the microtubule organizing center (MTOC), we visually examined certain MTOC components in the Δ*scp3* strain. As shown in Figs. 7 and 8A, the localization and intensity of the fluorescent signal of the three proteins, Alp4, Mto1 and Ase1, were visibly normal in the Δ*scp3* strain, further suggesting that MT misorientation in this strain is due to the loss of bundling.

### The effect of CIPC in Δ*ase1*

Although CIPC did not disturb the orientation of MTs during mitosis in wild-type cells, we found that it did have an effect on the Δ*ase1* strain. As shown in Fig. 8B, Δ*ase1* cells were arrested around anaphase or cytokinesis in the presence of the drug and were unable to grow on YES medium containing the drug (Fig. 8C). However, the localization of Ase1 was not affected by CIPC (Fig. 8B). These results suggested that CIPC targets a protein that has a function that is redundant with Ase1.

In this study, we showed that CIPC affects the orientation of cytoplasmic MTs in the wild-type background of fission yeast. Although CIPC induces multipolar mitotic spindles in higher eukaryotes, it does not disturb cell cycle progression through mitosis or mitotic spindle morphology in fission yeast. This discrepancy suggests that 1) the target of the drug is different or 2) the organization of MTs is regulated by redundant mechanisms, one of which requires the target of CIPC. The dependency on each mechanism is likely to differ among the species.

The target of CIPC in fission yeast remains to be identified. It is possible that Scp3 or Cps3 is a target. Molecular cloning of the *cps3* ^+^ gene followed by functional analyses with other genes would lead a conclusion in this regard. Although CIPC causes cell lethality at 300 μM, *scp3* ^+^ or *ase1* ^+^ gene knockout does not, suggesting that the products of these two genes are not targets. We should, however, be cautious of concluding that the drug targets other proteins. Furthermore, CIPC might cause lethality by affecting multiple cellular processes. Supporting this notion, it has been reported that the *cps1* ^+^ and *cps8* ^+^ genes, the mutations of which both confer hypersensitivity to CIPC, encode proteins with 55% sequence identity to Fks1p or Fks2p, which are proposed to be catalytic or associated subunits of *Saccharomyces cerevisiae* 1,3-beta-D-glucan synthase [47,48] and actin [49,50], respectively.

## Supporting Information

S1 Fig Mitotic progression in cells treated with CIPC. (A) Cells were observed by time lapse microscopy at a two-minute interval. The bar indicates 10 μm. (B) The results shown in (A) was statistically analyzed by Nonparametric Mann-Whitney *U* test. The time for mitotic progression was defined by time from appearance of the mitotic spindle to disappearance (DMSO n = 28, 260 μM CIPC n = 38). The red and gray lines indicate the mean and the SD, respectively.
(PDF)

S2 Fig Characterization of Scp3-GFP. (A) Mixture of the wild type cells and cells expressing Scp3-GFP from the native promoter (left) or mixture of the wild type cells and cells expressing Scp3-GFP from the plasmid, pREP81-*scp3* ^+^-*gfp* (right) was observed for comparison of the intensity of the signal from Sad1-mCherry. Arrowheads indicate cells expressing Scp3-GFP. The bar is 5 μm. (B) Microtubules were observed in each strain. (C) Nonparametric Mann-Whitney *U* test of (B) for analysis of MT-orientation. More than 150 microtubules were observed for each condition. The red lines are the median. (D) Each strain was grown on YES medium with DMSO or 260 μM CIPC at 30°C for 5 days.
(PDF)

S3 Fig Phenotype of double mutant, Δ*ase1* Δ*scp3*. (A) Microtubules were observed in each strain. The bar is 5 μm. (B) Nonparametric Mann-Whitney *U* test of (A) for analysis of MT-orientation. More than 150 microtubules were observed for each condition. The red lines are the median.
(PDF)
