# Supplementary figures and images for: Fission Yeast Scp3 Potentially Maintains Microtubule Orientation through Bundling
Source: PLoS One. 2015 Mar 13;10(3):e0120109. doi: 10.1371/journal.pone.0120109 (PMC4359140; doi:10.1371/journal.pone.0120109)

A

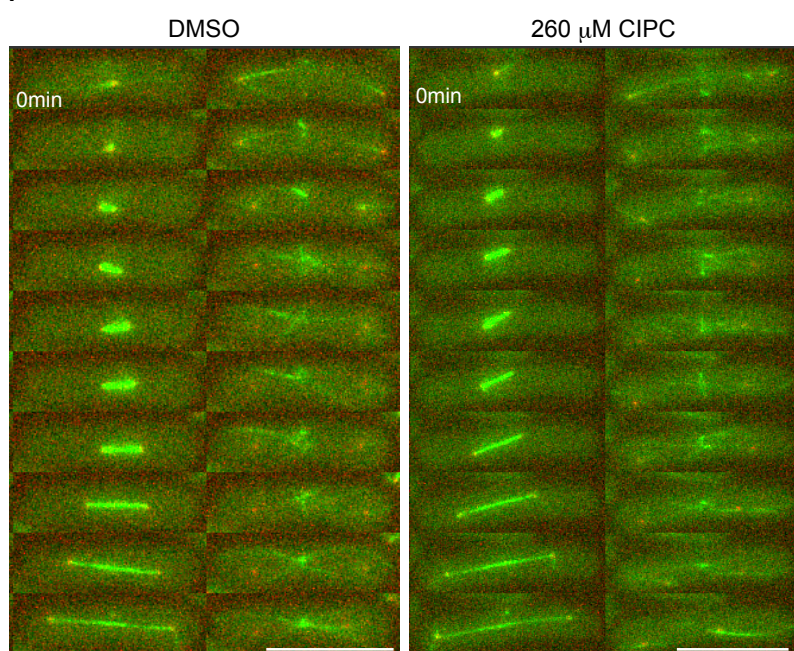

B

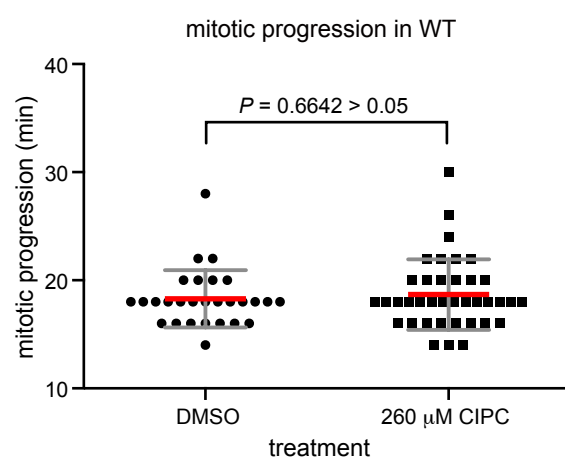

Supplement: S1 Fig — (A) Cells were observed by time lapse microscopy at a two-minute interval. The bar indicates 10 μm. (B) The results shown in (A) was statistically analyzed by Nonparametric Mann-Whitney U test. The time for mitotic progression was defined by time from appearance of the mitotic spindle to disappearance (DMSO n = 28, 260 μM CIPC n = 38). The red and gray lines indicate the mean and the SD, respectively. (PDF) [file pone.0120109.s001.pdf]

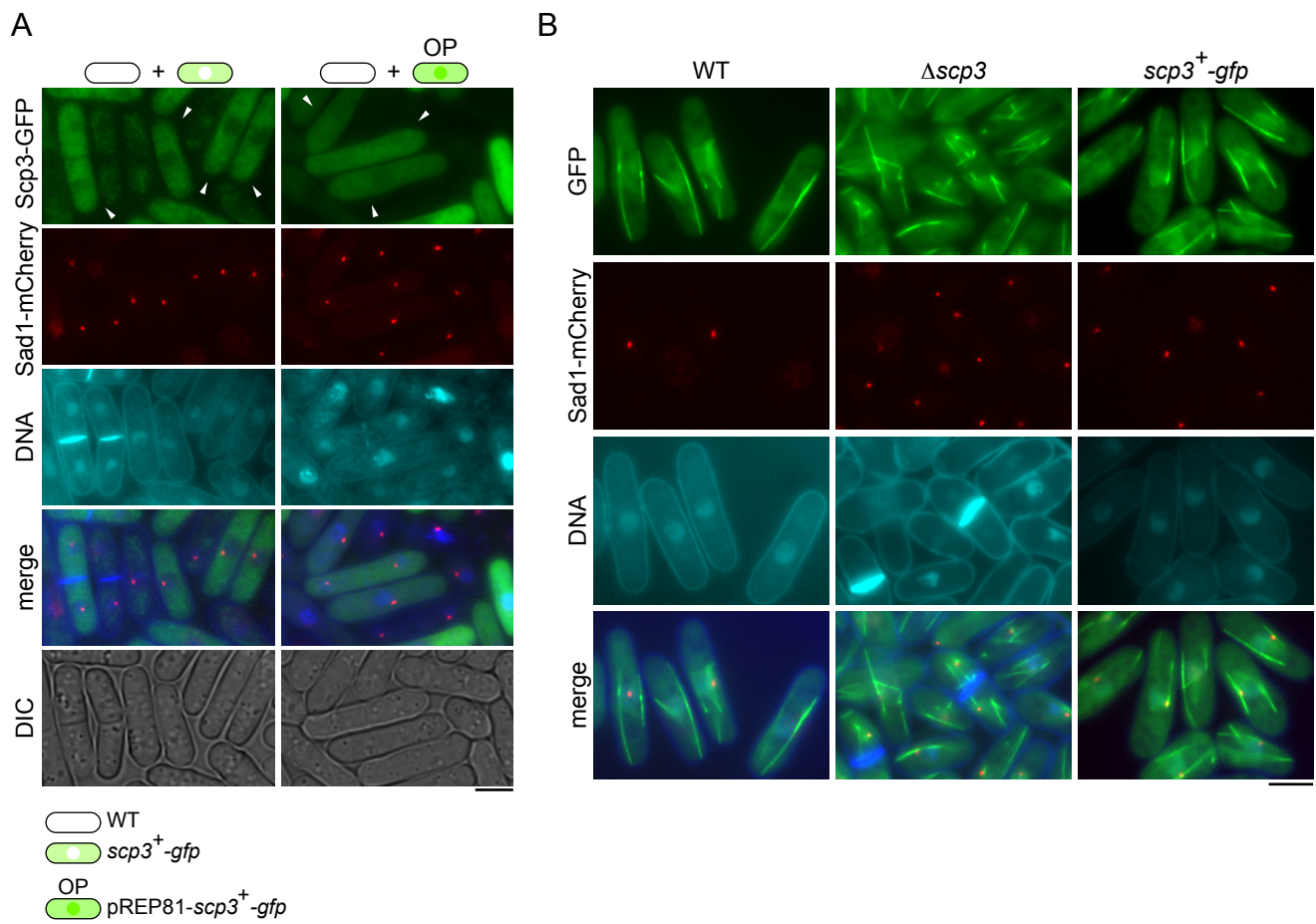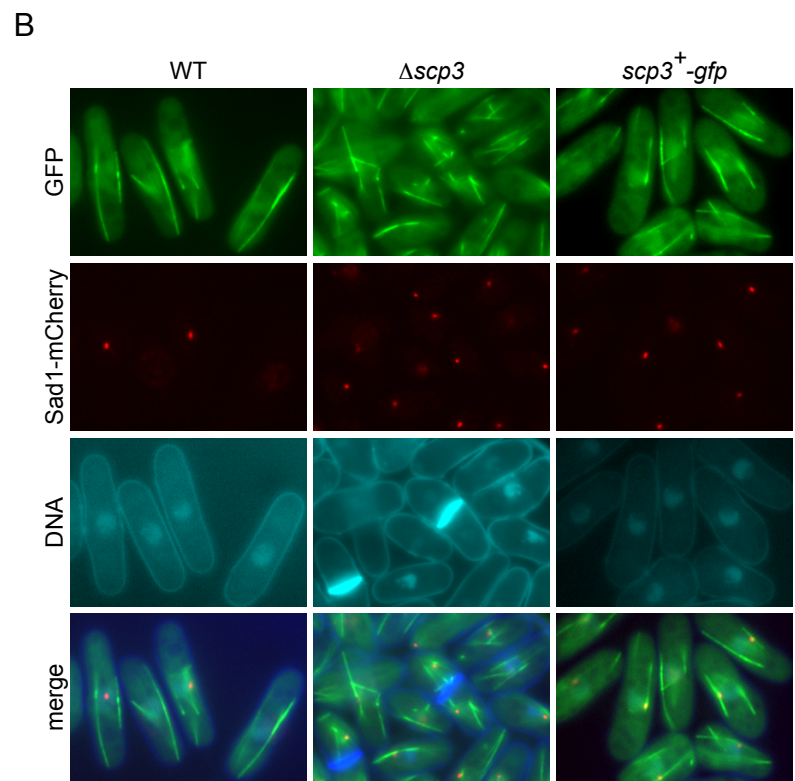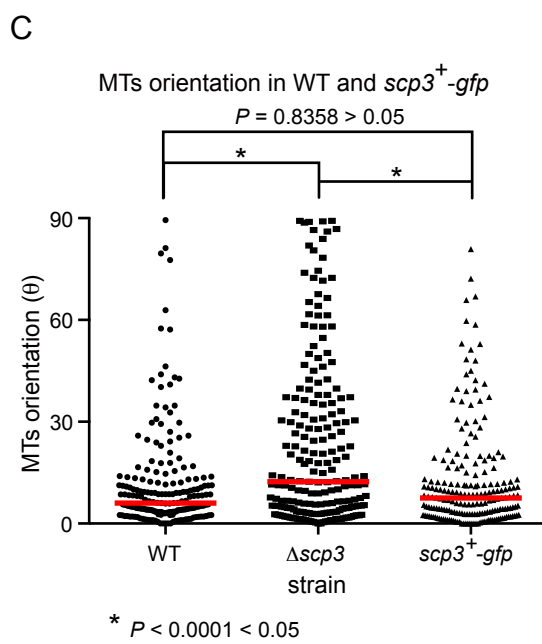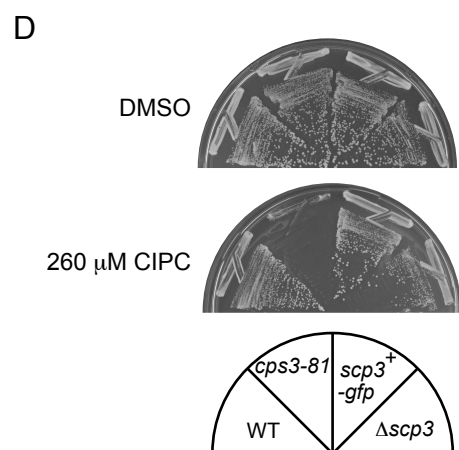

Supplement: S2 Fig — (A) Mixture of the wild type cells and cells expressing Scp3-GFP from the native promoter (left) or mixture of the wild type cells and cells expressing Scp3-GFP from the plasmid, pREP81-scp3 +-gfp (right) was observed for comparison of the intensity of the signal from Sad1-mCherry. Arrowheads indicate cells expressing Scp3-GFP. The bar is 5 μm. (B) Microtubules were observed in each strain. (C) Nonparametric Mann-Whitney U test of (B) for analysis of MT-orientation. More than 150 microtubules were observed for each condition. The red lines are the median. (D) Each strain was grown on YES medium with DMSO or 260 μM CIPC at 30°C for 5 days. (PDF) [file pone.0120109.s002.pdf]

A

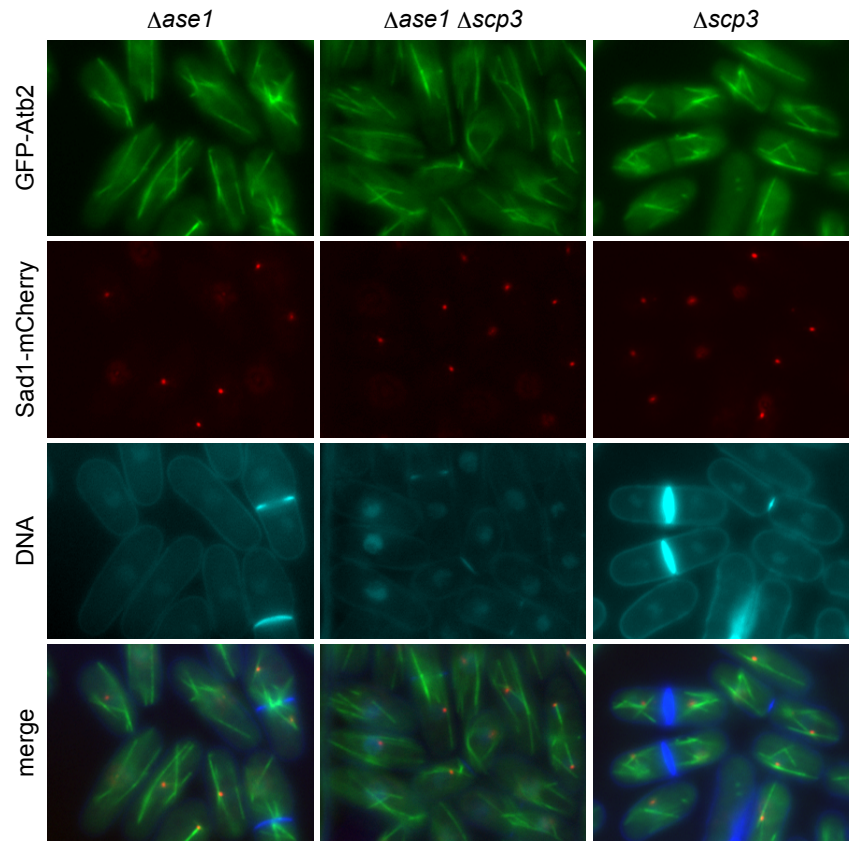

B

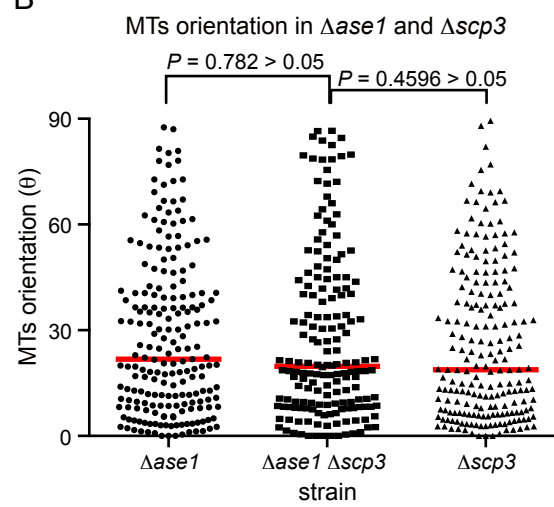

Supplement: S3 Fig — (A) Microtubules were observed in each strain. The bar is 5 μm. (B) Nonparametric Mann-Whitney U test of (A) for analysis of MT-orientation. More than 150 microtubules were observed for each condition. The red lines are the median. (PDF) [file pone.0120109.s003.pdf]
